# Myeloid liver kinase B1 contributes to lung inflammation induced by lipoteichoic acid but not by viable *Streptococcus pneumoniae*

**DOI:** 10.1186/s12931-022-02168-6

**Published:** 2022-09-12

**Authors:** Liza Pereverzeva, Natasja A. Otto, Joris J. T. H. Roelofs, Alex F. de Vos, Tom van der Poll

**Affiliations:** 1 Center for Experimental and Molecular Medicine, Amsterdam University Medical Centers, Location Academic Medical Center, University of Amsterdam, Meibergdreef 9, G2-129, 1105 AZ Amsterdam, The Netherlands; 2 Amsterdam Infection & Immunity Institute, Amsterdam, The Netherlands; 3 Department of Pathology, Amsterdam University Medical Centers, Amsterdam, The Netherlands; 4 Amsterdam Cardiovascular Sciences, Amsterdam University Medical Centers, Amsterdam, The Netherlands; 5 Division of Infectious Diseases, Amsterdam University Medical Centers, Location Academic Medical Center, University of Amsterdam, Amsterdam, The Netherlands

**Keywords:** Liver kinase B1, *Streptococcus pneumoniae*, Lipoteichoic acid, Pneumonia, Alveolar macrophages

## Abstract

**Background:**

Liver kinase B1 (Lkb1, gene name *Stk11*) functions as a tumor suppressor in cancer. Myeloid cell Lkb1 potentiates lung inflammation induced by the Gram-negative bacterial cell wall component lipopolysaccharide and in host defense during Gram-negative pneumonia. Here, we sought to investigate the role of myeloid Lkb1 in lung inflammation elicited by the Gram-positive bacterial cell wall component lipoteichoic acid (LTA) and during pneumonia caused by the Gram-positive respiratory pathogen *Streptococcus pneumoniae* (*Spneu*).

**Methods:**

Alveolar and bone marrow derived macrophages (AMs, BMDMs) harvested from myeloid-specific Lkb1 deficient (*Stk11*-ΔM) and littermate control mice were stimulated with LTA or *Spneu *in vitro. *Stk11*-ΔM and control mice were challenged via the airways with LTA or infected with *Spneu *in vivo.

**Results:**

Lkb1 deficient AMs and BMDMs produced less tumor necrosis factor (TNF)α upon activation by LTA or *Spneu.* During LTA-induced lung inflammation, *Stk11*-ΔM mice had reduced numbers of AMs in the lungs, as well as diminished cytokine release and neutrophil recruitment into the airways. During pneumonia induced by either encapsulated or non-encapsulated *Spneu*, *Stk11*-ΔM and control mice had comparable bacterial loads and inflammatory responses in the lung*,* with the exception of lower TNFα levels in *Stk11*-ΔM mice after infection with the non-encapsulated strain.

**Conclusion:**

Myeloid Lkb1 contributes to LTA-induced lung inflammation, but is not important for host defense during pneumococcal pneumonia.

**Supplementary Information:**

The online version contains supplementary material available at 10.1186/s12931-022-02168-6.

## Background

Lower respiratory tract infections remain in the top ten of mortality causes worldwide [1], with *Streptococcus pneumoniae* (*Spneu*) accounting for the most common bacterial pathogen of community-acquired pneumonia [2]. When pneumococci enter the lower respiratory tract, alveolar macrophages (AMs) are first in line to capture the bacteria and initiate a host response [3]. However, invasive strains of these Gram-positive bacteria are characterized by a thick polysaccharide capsule that helps the organism invade the lung and escape the immune system [2]. This raises the interest to study the function of AMs during the host response to *Spneu*, exploring new potential targets for the treatment of pneumonia.

Over the last few years it has become evident that Liver kinase B1 (Lkb1) impacts the performance of macrophages during the immune response [4–7]. Lkb1, also known as serine/threonine kinase 11 (STK11), plays a major role in many cell processes such as proliferation and development [8–10], and cell metabolism [11]. It was first recognized as a tumor suppressor gene in Peutz-Jeghers Syndrome [12], and is now known to be involved in many other malignancies [13]. In the field of immune responses to infectious pathogens, Lkb1 has been described to have a suppressive effect on the pro-inflammatory activity of macrophages [5, 7] and to be involved in the proliferation of AMs [7]. Two recent studies, including one from our group, reported that lack of Lkb1 in the myeloid lineage of mice is associated with reduced numbers of AMs [6, 7], which was accompanied by an impaired antibacterial defense during pneumonia caused by *Klebsiella (K.) pneumoniae* [6] or *Staphylococcus (S.) aureus* [7]. Myeloid Lkb1 deficiency resulted in exaggerated lung pathology during *S. aureus*, but not during *Klebsiella* pneumonia [6, 7], suggesting that the role of this protein in the host response during lower respiratory tract infection at least in part depends on the causative pathogen. In this respect it is important to note that myeloid Lkb1 deficient mice demonstrated strongly reduced cytokine release in the airways upon intrapulmonary delivery of lipopolysaccharide (LPS), a major component of Gram-negative bacteria (including *Klebsiella*) and a potent Toll-like receptor (TLR)4 agonist [6].

Here, we sought to investigate the role of myeloid Lkb1 in lung inflammation induced by the Gram-positive bacterial wall component and TLR2 agonist lipoteichoic acid (LTA) [14] and viable *Spneu*. For this we used myeloid-specific Lkb1 deficient mice and well-established mouse models of an airway LTA challenge and pneumococcal pneumonia. Our research suggests that Lkb1 plays a role in the TLR2-mediated inflammatory response of myeloid cells, but that its potential function is obscured during respiratory infection by viable pneumococci.

## Methods

### Animals

Homozygous *Stk11*^fl/fl^ mice (014143; Jackson Laboratory, Bar Harbor, ME) [10] were crossed with LysM^cre^ mice [15] to generate myeloid cell specific Lkb1-deficient (*Stk11*-ΔM) mice. *Stk11*^fl/fl^ cre-negative littermates were used as controls in all experiments. All genetically modified mice were backcrossed at least 6 times to a C57Bl/6 background and housed under standard care. Mice were age and sex matched and used in experiments at 8–12 weeks of age. Experiments were performed in accordance with the Dutch Experiment on Animals Act and approved by the Central Commission for Animal Experiments.

### Bone-marrow isolation and differentiation

Bone-marrow derived macrophages (BMDMs) were obtained by harvesting bone marrow from tibias and femurs of naïve mice by flushing with sterile phosphate buffered saline (PBS; Invitrogen, Carlsbad, California). Clumps were removed by dispersing the cells using a syringe with a 21G needle. Cells were spun down at 1250 rpm for 5 min at 4 °C. Cells were suspended in complete medium (RPMI 1640 with l-glutamine and 25 mM HEPES; Gibco, Thermo Fisher, Waltham, MA) containing 10% fetal bovine serum and 1% penicillin/streptomycin supplemented with 15% L929-conditioned medium (as source of M-CSF; produced as described in [16]) and cultured at 37 °C and 5% CO_2_ to differentiate into BMDMs. After 7 days of differentiation, adherent BMDMs were washed with PBS and detached with trypsin (Lonza, Basel, Switzerland). Cells were seeded in 48-wells flat bottom culture pates (Greiner Bio-one Frickenhausen, Germany) at a density of approximately 250,000 cells per well in complete medium and left to adhere overnight.

### Isolation of alveolar macrophages

Naive mice were anesthetized with isoflurane and terminated by cervical dislocation. AMs were harvested by bronchoalveolar lavage (BAL) with PBS containing 2 mM ethylenediaminetetraacetic acid (EDTA). Cells were seeded in 96-wells flat bottom culture pates (Greiner Bio-one Frickenhausen, Germany) at a density of approximately 40,000 cells per well in complete medium and left to adhere overnight.

### Cell stimulation

Adherent BMDMs and AMs were washed with PBS, and stimulated for 4 or 24 h with 10 µg/ml ultrapure LTA (from *S. aureus*, Invivogen, San Diego, CA), heat-killed *Spneu* 6303 (ATCC 6303, American Type Culture Collection, Manassas, VA) at a multiplicity of infection (MOI) 10:1, a non-encapsulated mutant strain of *Spneu* D39 (isogenic capsule locus (cps) deletion mutant *D39Δcps*) [17] MOI 100:1, or medium control. After stimulation, supernatant for cytokine measurements was stored at − 20 °C and analyzed as described beneath. Viability of BMDMs was assessed after 24 h of stimulation in non-adherent polypropylene 96-wells plates (Greiner Bio-One, Kremsmünster, Austria). Cells were washed with PBS, stained with fixable viability dye eFluor 780 (Invitrogen) and analyzed by flow cytometry as outlined beneath.

### Lung inflammation model

Lung inflammation in mice was induced by intranasal administration of 100 µg of ultrapure LTA (*S. aureus*, Invivogen, San Diego, CA) in 50 µl normal saline as previously described [18, 19]. Six hours after LTA installation, mice were euthanized as described above and BAL was performed with 5 × 500 µl sterile PBS containing 2 mM EDTA (Invitrogen, Carlsbad, CA). BAL fluid (BALF) was stored at − 20 °C until analysis; cells were analyzed by flow cytometry. Lungs were digested as described before [6] and stained for analysis by flow cytometry.

### Mouse infection model

Pneumonia was induced by intranasal inoculation with approximately 5 × 10^4^ colony forming units (CFU) of *Spneu* serotype 3 (ATCC 6303) or 1 × 10^8^ CFU of *Spneu D39Δcps*. Infection and processing of organs were done as described elsewhere [20]. In brief, mice were euthanized at 12 or 40 h after infection with *Spneu* 6303 for collection of blood, lungs, spleens and livers. Tissue was homogenized or fixed for histopathology (lungs). *Spneu D39Δcps*-infected mice were euthanized after 5 h of infection for collection of BALF and lungs (for homogenization). Bacterial loads were determined by counting CFU from serial dilutions plated on blood agar plates, incubated at 37 °C for 16 h. Lung homogenates were made exactly as described previously [21]. Briefly, lung material was collected in 4 volumes of cold (4 °C) sterile saline and homogenized for 10 s using a tissue homogenizer (Qiagen). For cytokine and chemokine measurements, lung homogenates were lysed in an equal volume of lysis buffer (150 mM NaCl, 15 mM Tris, 1 mM MgCl(H_2_O)_6_, 1 mM CaCl_2_(H_2_O)_2_, 1% Triton, pH 7.4) with protease inhibitor (Roche Complete Protease Inhibitor cocktail) on ice for 30 min. Lysates were then spun down; supernatants were stored at − 20 °C until analysis.

### Flow cytometry

Total cell counts in BALF and lung digestions were assessed by flow cytometry using Precision Count Beads™ (BD Bioscience, San Jose, CA). Cell subsets were identified by staining with fixable viability dye eFluor 780 (Invitrogen) and the following antibodies: rat anti-mouse CD45 PE-eFluor610 (clone 30-F11), hamster anti-mouse CD11c PerCP-Cy5,5 (clone HL3), rat anti-mouse CD11b PE-Cy7 (clone M1/70), rat anti-mouse Siglec-F AlexaFluor647 (clone E50-2440), rat anti-mouse Ly-6G AlexaFluor700 (clone 1A8) (all from BD Biosciences); mouse anti-mouse CD64 PerCP-Cy5,5 (clone X54-5/7.1), rat anti-mouse MerTK PE (clone 2B10C42), rat anti-mouse Ly-6G FITC (clone 1A8) (all from Biolegend, San Diego, CA). Flow cytometry was performed using a CytoFLEX S (Beckman Coulter) and data were analyzed using FlowJo software (BD Biosciences). Gating of cell populations was performed as described previously [6]. Neutrophils were gated on CD11c^neg^Ly6G^pos^ cells.

### RNA isolation and transcription analysis

Total RNA was extracted from BMDMs using the Nucleospin RNA isolation kit (Marcherey-Nagel, Düren, Germany) following the manufacturer’s instructions. Reverse transcription was performed using the M-MLV Reverse Transcriptase (Promega, Madison, WI) in the presence of RNase inhibitor (ThermoFisher, Waltham, MA) with 300 ng of DNase I (Roche, Basel, Switzerland) treated total RNA. RT-PCR was performed on LightCycler 480 (Roche, Basel, Switzerland) using the SensiFAST SYBR No-ROX Kit (Bioline, London, UK). Gene expression was normalized to HPRT as a housekeeping gene.

### Assays

Interleukin (IL)-1β, IL-6, IL-10, tumor necrosis factor α (TNFα), C-X-C Motif Chemokine Ligand (CXCL)1, CXCL2 and myeloperoxidase (MPO) were measured by ELISA according to the manufacturers protocol (R&D Systems, Minneapolis, MN).

### Histopathology

Pathology was performed exactly as described [21]. Briefly, one lung lobe of each mouse was carefully harvested by cutting the bronchia with a scissor, placed in a pathology cassette and fixed in standard 10% formaldehyde (i.e. 4% paraformaldehyde) for 24 h at room temperature and embedded in paraffin. Four-micrometer sections of the lung were stained with hematoxylin and eosin and scored by an independent pathologist as described elsewhere [20]. The following parameters were scored on a scale of 0 (absent), 1 (mild), 2 (moderate), 3 (severe), and 4 (very severe): interstitial inflammation, endothelitis, bronchitis, edema, thrombus formation, and pleuritis. In all experiments, the samples were scored by the same pathologist blinded for experimental groups.

### Statistical analysis

Non-parametric variables were analyzed using the Mann–Whitney U test. Parametric variables were analyzed using Student’s t-tests for 2-group comparisons and multiple t-tests for 2-group comparisons with > 2 conditions. Analysis were done using GraphPad Prism version 9.1.0 (GraphPad Software, San Diego, CA). Statistical significance is shown as *P < 0.05, **P < 0.01, ***P < 0.001 or ****P < 0.0001.

## Results

### Lkb1-deficiency in macrophages is associated with impaired TNFα production upon stimulation with *Spneu* or LTA

**Fig. 1:**
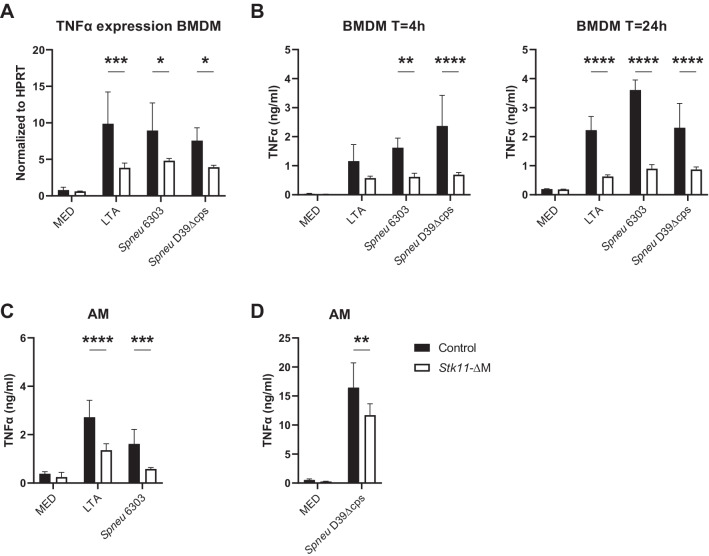
Lkb1-deficiency in macrophages is associated with decreased expression and production of TNFα upon stimulation with LTA or pneumococci. Bone marrow-derived macrophages (BMDMs) and alveolar macrophages (AMs) were harvested and stimulated with LTA (10 μg/ml), heat-killed *Streptococcus pneumoniae* (*Spneu*) 6303 (MOI 10:1) and uncapsulated *Spneu* D39 (*D39Δcps*) (MOI 100:1), or medium control. mRNA levels of TNFα (normalized to the housekeeping gene HPRT) in BMDMs after 4 h stimulation (**A**). TNFα production by BMDMs after 4 h and 24 h stimulation (**B**). TNFα production by AMs after 24 h stimulation with LTA and *Spneu* 6303 (**C**) and *Spneu D39Δcps* (**D**). Data are shown as bar graphs with mean ± SD representing technical replicates (n = 4 for BMDMs and n = 6 for AMs per condition). Gene expression and cytokine levels of macrophages from *Stk11-*ΔM mice were compared to littermate control mice using multiple t-test. *P < 0.05; **P < 0.01, ***P < 0.001, ****P < 0.0001

To investigate the role of Lkb1 in macrophages during activation by *Spneu*, we generated myeloid-specific Lkb1-deficient (*Stk11*-ΔM) mice by crossing Lkb1-floxed mice (*Stk11*^fl/fl^) with LysM^cre^ mice. A previous study by our group confirmed very low Lkb1 protein expression in BMDMs and AMs of *Stk11*-ΔM mice compared to littermate controls [6]. The ability to initiate a proper immune response by Lkb1-deficient macrophages was studied by in vitro stimulation of BMDMs and AMs with different strains of heat-killed pneumococci and the Gram-positive bacterial wall component LTA. We focused on TNFα since this proinflammatory cytokine is readily produced by AMs upon stimulation with *Spneu* [22, 23] and plays a major role in host defense during pneumonia caused by this pathogen [24]. Lkb1 deficient BMDMs exposed to LTA, the encapsulated *Spneu* 6303 or the unencapsulated *Spneu D39Δcps* showed reduced TNFα mRNA levels (4-h incubation) as compared to controls BMDMs (Fig. 1A). TNFα protein release by *Stk11*-ΔM BMDMs was also decreased after 4-h stimulation with *Spneu* 6303 or *Spneu D39Δcps*, and after 24 h for all conditions (Fig. 1B). Because Lkb1 deficiency has been associated with increased apoptosis [7], we sought to establish that the effect of reduced TNFα production was not due to impaired viability of *Stk11*-ΔM BMDMs. To enable measurement of cell viability by flow cytometry, we cultured BMDMs in non-adherent plates and stained them with a fixable viability dye. After stimulation with either LTA, *Spneu* 6303 or *Spneu D39Δcps*, the percentage live BMDMs was close to 100% and similar between genotypes (Additional file 1: Fig. S1A). We confirmed that the non-adherent condition did not alter the phenotype of Lkb1-deficient BMDMs, as they also showed impaired TNFα secretion (Additional file 1: Fig. S1B).

In two separate experiments, we studied the role of Lkb1 in TNFα production by AMs in response to LTA and *Spneu* 6303 (Fig. 1C), and *Spneu D39Δcps* (Fig. 1D). Lkb1-deficient AMs were impaired in their ability to produce TNFα in all mentioned conditions when compared with control AMs. Altogether, these results suggest that Lkb1 potentiates TNFα production by BMDMs and AMs upon activation by *Spneu*.

### *Stk11*-ΔM mice show reduced cytokine release and neutrophil influx in the lungs after LTA challenge

**Fig. 2:**
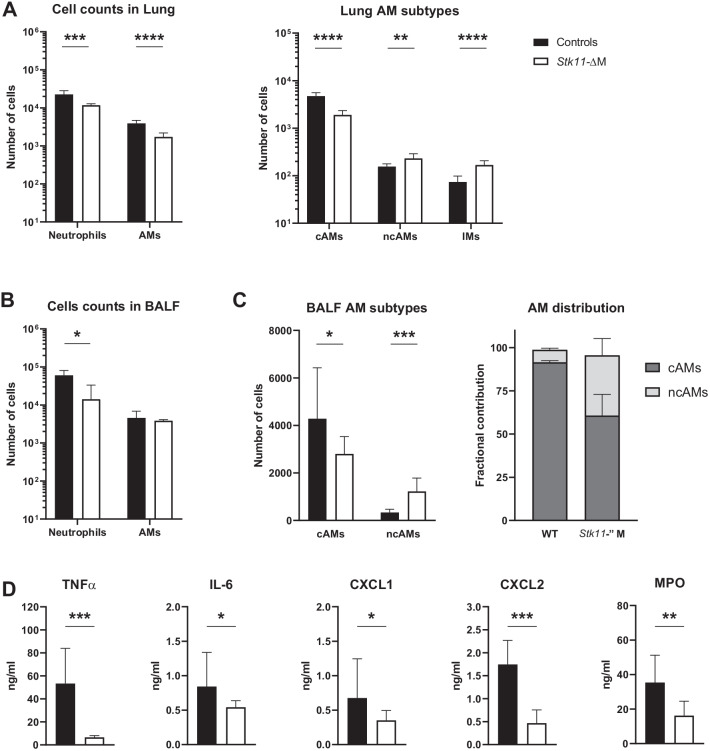
Macrophage Lkb1-deficient mice show reduced neutrophil influx and cytokine release into the airways on LTA challenge. Cell counts and phenotypes in lungs (**A**), BALF (**B**, **C**) and cytokine concentrations in BALF 6 h after intranasal inoculation with 100 μg LTA. Numbers of neutrophils and alveolar macrophages (AMs) in the lung (**A**) and BALF (**B**) after LTA challenge. Neutrophils were gated on CD11c^neg^Ly6G^pos^ cells. AM subtypes: “classic” AMs (cAMs) (CD11c^pos^SiglecF^high^CD11b^neg^), “non-classic” AMs (ncAMs) (CD11c^pos^SiglecF^low^CD11b^pos^) and interstitial macrophages (IMs) (SiglecF^neg^ and CD11b^high^). **C** Total number and fractional contribution of cAMs and ncAMs in BALF. **D** Cytokine and chemokine concentrations in BALF. Data are shown as bar graphs showing mean ± SD, representing 8 mice per group. Cell counts and cytokine levels of the *Stk11*-ΔM mice were compared to littermate controls using multiple student t-test for cells counts (**A**–**C**) and the Mann–Whitney U test for cytokine levels (**D**). *P < 0.05; **P < 0.01, ***P < 0.001, ****P < 0.0001

To investigate whether the role of Lkb1 in AMs would also stand during an acute inflammatory response in vivo, we administered LTA via the airways of *Stk11*-ΔM and control mice and studied the cell composition in BALF and the lung, as well as cytokine and chemokine levels in BALF (Fig. 2). Previous results from our group showed a decreased number of AMs in the lungs of naive *Stk11*-ΔM mice, which was explained by lower numbers of ‘classical’ (CD11c^pos^SiglecF^high^CD11b^neg^) AMs (cAMs) [6]. We were therefore interested to investigate the pulmonary cell composition after an inflammatory challenge in these mice. In agreement with our findings in naïve mice [6], LTA challenged *Stk11*-ΔM mice had lower AMs counts in whole lung cell suspensions, caused by reduced cAM numbers (Fig. 2A). As a possible compensatory mechanism, LTA administered *Stk11*-ΔM mice had higher numbers of “non-classical” (CD11c^pos^SiglecF^low^CD11b^pos^) AMs (ncAMs) and interstitial macrophages (IMs), but this did not restore total AM numbers to those in control mice. In addition, *Stk11*-ΔM mice had a significantly reduced neutrophil influx (CD11c^neg^Ly6G^pos^ cells) into the lung compared to control mice 6 h after LTA inoculation (Fig. 2A). Important to note is that the lungs were digested after BAL was performed, indicating that these numbers represent cells residing in lung tissue. In BALF, neutrophil influx was also impaired in *Stk11*-ΔM mice, while AM counts were similar to those in control mice (Fig. 2B). Akin to results from lung tissue, cAM numbers were higher and ncAM counts lower in BALF from *Stk11*-ΔM mice (Fig. 2C). Moreover, LTA challenged *Stk11*-ΔM mice had significantly lower levels of cytokines (TNFα, IL-6) and chemokines (CXCL1, CXCL2), as well as MPO concentrations, in BALF compared to control mice (Fig. 2D). These data suggest that macrophage Lkb1 contributes to LTA-induced lung inflammation.

### *Stk11*-ΔM mice show an unaltered response during pneumonia caused by encapsulated *Spneu*

**Fig. 3:**
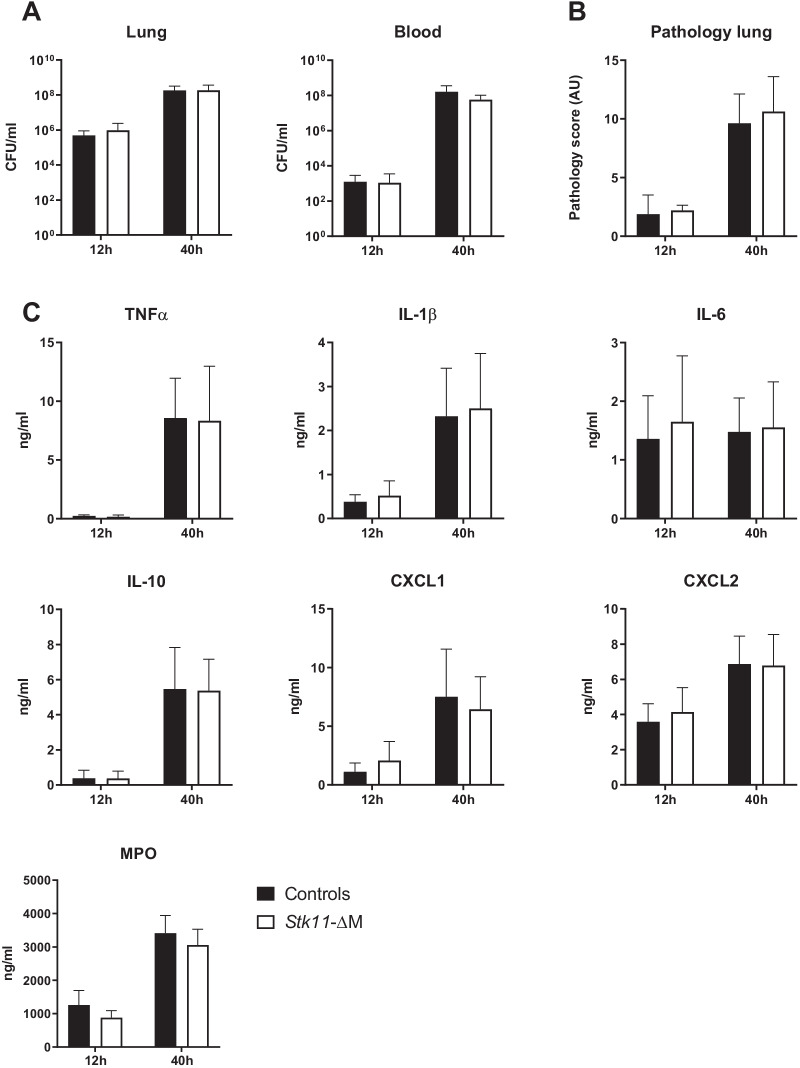
Macrophage Lkb1 does not play a role in the host defense during pneumonia caused by encapsulated *S. pneumoniae*. Mice were inoculated intranasally with approximately 5 × 10^4^ colony-forming units (CFUs) of encapsulated *Spneu* 6303 and euthanized 12 and 40 h thereafter for determination of bacterial loads (CFUs per milliliter) in the lung and blood (**A**), the extent of lung inflammation scored on haematoxylin and eosin stained tissue sections as total pathology score (**B**), and cytokine and chemokine levels (TNFα, IL-1β, IL-6, IL-10, CXCL1, CXCL2 and MPO) in the lung (**C**). Data are shown as bar graphs with mean ± SD representing 6–8 mice per group at each time point. Groups were compared using the Mann–Whitney U test. All comparisons were not significant

We next sought to determine the role of myeloid cell Lkb1 in the host response during pneumonia caused by viable pneumococci. To this end, we infected *Stk11*-ΔM and control mice with *Spneu* 6303 and measured bacterial growth and dissemination, and lung inflammatory reactions at 12 and 40 h after infection. Remarkably, Lkb1 deficiency in myeloid cells did not influence any of the responses, as illustrated by comparable bacterial outgrowth in lungs and distant organs (Fig. 3A and Additional file 2: Fig. S2), and similar lung pathology scores (Fig. 3B) and pulmonary cytokine levels (Fig. 3C).

### Impaired TNFα release in the lung of *Stk11*-ΔM mice during pneumonia with non-encapsulated *Spneu*

**Fig. 4:**
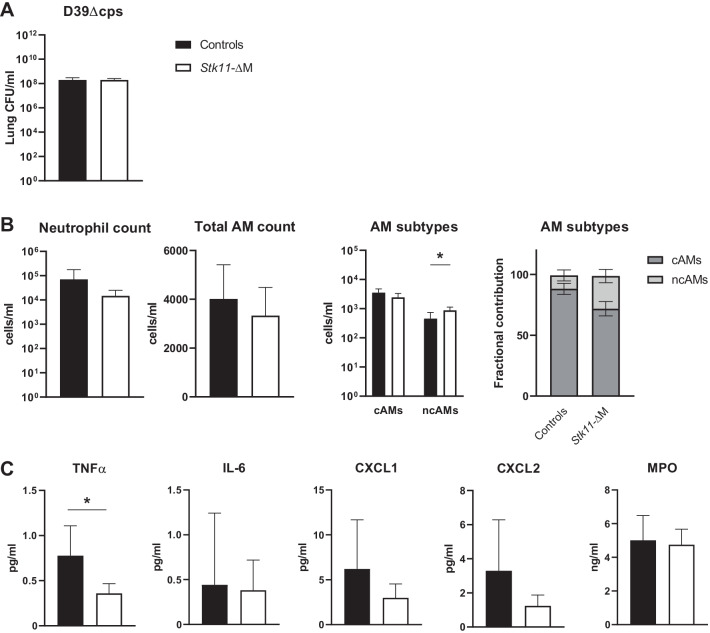
Macrophage Lkb1-deficient mice have a reduced capacity to produce TNFα in the lung upon infection with non-encapsulated *S. pneumoniae*, whilst bacterial outgrowth and neutrophil influx are unaltered. Mice were inoculated intranasally with approximately 1 × 10^8^ colony-forming units (CFUs) of *Spneu D39Δcps* and euthanized 5 h thereafter for determination of bacterial loads (CFUs per milliliter) in the lung (**A**), total number of neutrophils (CD11c^neg^Ly6G^pos^) and alveolar macrophages (AMs), and fractional contribution of “classic” AMs (cAMs) (CD11c^pos^SiglecF^high^CD11b^neg^) and “non-classic” AMs (ncAMs) (CD11c^pos^SiglecF^low^CD11b^pos^) in BALF (**B**) and cytokine and chemokine (TNFα, IL-6, CXCL1, CXCL2 and MPO) levels in BALF (**C**). Data are shown as bar graphs with mean ± SD representing 7 mice per group. Bacterial loads (**A**) and cytokine levels (**C**) of *Stk11*-ΔM mice were compared to littermate controls using the Mann–Whitney U test, and cells counts (**B**) were compared by student’s t-test. *P < 0.05

The capsule of *Spneu* is a major virulence factor, shielding the pathogen from the host immune system [2]. Our group previously showed that part of the virulence of encapsulated pneumococci depends on the capacity of the capsule to impede recognition of TLR ligands expressed by this bacterium [17]. Considering that *Spneu* 6303 has a particularly thick capsule [25], we hypothesized that a role of myeloid cell Lkb1 would be exposed after infection with a unencapsulated *Spneu* strain. Therefore, we infected *Stk11*-ΔM and control mice with viable *D39Δcps* and compared their responses. Myeloid Lkb1-deficiency did not affect *Spneu D39Δcps* counts in the lungs 5 h after infection (Fig. 4A). Furthermore, neutrophil influx (CD11c^neg^Ly6G^pos^ cells) and total AM counts in BALF were similar between *Stk11*-ΔM and control mice (Fig. 4B). However, in line with the findings in the LTA-inflammation model, the proportion of ncAMs was higher in *Stk11*-ΔM mice. The levels of TNFα were lower in BALF (Fig. 4C) and lungs (Additional file 3: Fig. S3) of *Stk11*-ΔM compared to control mice, whilst levels of IL-6, CXCL1, CXCL2 and MPO were similar between groups. These results suggest that myeloid cell Lkb1 only plays a role in TNFα production after infection with unencapsulated *Spneu*, while all inflammatory responses do not rely on Lkb1.

## Discussion

Lkb1 has been widely studied as a tumor suppressor gene in the context of cancer. Here we used myeloid-specific Lkb1 deficient mice to demonstrate that Lkb1 plays a role in the TLR2-mediated inflammatory response. In two types of macrophages, AMs and BMDMs, loss of Lkb1 was associated with reduced TNFα production upon stimulation with LTA and *Spneu*. Moreover, during lung inflammation upon LTA challenge, mice lacking Lkb1 in myeloid cells had decreased AM numbers and lower cytokine levels in the lungs. However, myeloid Lkb1 appeared not to play a role in the host response during respiratory infection caused by viable pneumococci.

Earlier studies on the role of Lkb1 in the inflammatory response of macrophages focused on effects of the Gram-negative bacterial component LPS, a TLR4 agonist [4–6, 26]. To our best knowledge the involvement of Lkb1 in the TLR2-mediated response specific for Gram-positive bacteria has remained unexplored. In the current study, we expand existing evidence that the role of Lkb1 in pneumonia is pathogen specific. A previous study from our group documented that myeloid Lkb1-deficiency results in reduced numbers of AMs in the lung, and that Lkb1 deficient AMs had an unaltered TNFα production upon in vitro exposure to LPS or the Gram-negative pathogen *K. pneumoniae* [6]. Another investigation reported that Lkb1 inhibits LPS-induced NF-κB activation, resulting in higher TNFα production by Lkb1-deficient BMDMs [5]. Contrarily, our current data show that upon in vitro stimulation of AMs with the Gram-positive bacterial component LTA (a TLR2 agonist) [14], Lkb1 is required for adequate TNFα production. This role of Lkb1 in response to LTA-induced cytokine production was confirmed in another type of macrophages (BMDMs), and by stimulation with the Gram-positive bacterium *Spneu*.

During lung inflammation caused by a LTA challenge via the airways, *Stk11*-ΔM mice had decreased cytokine release (including IL-6) and a reduced neutrophil influx into the lungs. In the early phase of a proinflammatory response to invasive pathogens in the airways, neutrophil recruitment into the lung is generally orchestrated by AMs [27]. During LTA-induced lung inflammation, neutrophil influx occurs in a TLR2-dependent manner [28]. Reduced numbers of AMs in the lungs of *Stk11*-ΔM mice, as well as the impaired function of Lkb1-deficient AMs as determined in the in vitro model, could explain the decreased recruitment of neutrophils, along with the lower levels of the neutrophil degranulation product MPO in BALF of *Stk11*-ΔM mice. Of note, our group previously showed that neutrophil influx was not impaired in *Stk11*-ΔM mice challenged with the TLR4 agonist LPS, which was accompanied by unaffected IL-6 levels [6]. Together these data suggest that neutrophil recruitment upon airway administration of LTA may partially be mediated by AMs and TLR2-Lkb1 mediated IL-6 production [29].

Upon infection with the non-encapsulated *Spneu* strain, neutrophil influx into the lung was not significantly impaired in myeloid Lkb1-deficient mice, and MPO levels in BALF were comparable to control mice. This implicates that the presence of pneumococci in the lung, independently of their thick capsule, leads to neutrophil recruitment by means other than TLR2-Lkb1 mediated signalling in AMs. Apart from its capsule, pneumococci carry other important virulence factors, such as pneumolysin [2]. Whilst *Spneu* is primarily recognized by TLR2 [2, 23], TLR4 has been implicated as the receptor for pneumolysin and could therefore be a potential mediator of the neutrophil influx [30, 31]. Furthermore, detection of bacterial DNA from *Spneu* by TLR9 has been identified as an important receptor in the defence to pneumococci, as TLR9 deficient mice were highly susceptible to lethal infection [32]. Another explanation for the difference in Lkb1-mediated host response to LTA and *Spneu* could be a role of non-myeloid cells (not affected in *Stk11*-ΔM mice), considering that mice with a global TLR2 deficiency showed a reduced neutrophil influx during pneumococcal pneumonia [23]; airway epithelial cells may play a role in this context [33, 34].

In experiments using viable pneumococci, myeloid Lkb1 deficiency did not impact inflammatory responses with the exception of TNFα production after infection with the non-encapsulated *Spneu D39Δcps* strain. This finding taken together with the unaltered TNFα levels in *Stk11*-ΔM mice infected with the capsulated *Spneu* strain is in agreement with a previous in vivo investigation from our laboratory showing that during pneumonia the pneumococcal capsule can impede recognition of TLR ligands expressed by this bacterium [17].

A limitation of this study is the fact that the LTA used in the experiments was derived from *S. aureus* and not *Spneu*, while their structures differ in some significant ways [35]. Notably, however, even within different strains of *Spneu*, LTA structures and characteristics vary. Nonetheless, a study comparing LTA from *S. aureus* and two different *Spneu* strains, described no differences in important characteristics such as TLR2-dependence for TNF production [36]. Another limitation of our study is that we have not used highly purified LTA from surface lipoprotein-deficient (∆lgt) bacteria, but a preparation which may contain lipoproteins. Several studies have shown that lipoproteins, rather than LTA, are the actual bioactive TLR2 ligands in purified preparations of LTA [37–39]. Since both *Spneu* LTA and lipoproteins have previously been implicated in activation of TLR2 [40, 41], our experiments with LTA provide insight into the role of Lkb1 in the inflammatory response to TLR2 ligands. Further studies, however, with synthethic TLR2 ligands or LTA preparations from *Spneu∆lgt* are required to establish which bacterial ligands trigger Lkb1-dependent inflammatory responses.

## Conclusions

We here report that myeloid Lkb1 has an important role in the induction of TLR2 mediated lung inflammation. In contrast, its contribution to the host response during infection of the respiratory tract by viable pneumococci is highly limited. Taken together with our earlier study showing a strongly impaired antibacterial defense during pneumonia caused by *Klebsiella* [6], these results exemplify the complex nature of the innate immune response in the airways, triggered by an interaction between various pattern recognition receptors expressed by distinct host cell types and a variety of pathogen associated molecular patterns expressed by multiplying microorganisms.

## Supplementary Information

**Additional file 1: Figure S1.** Viability of in vitro stimulated Lkb1-deficient macrophages. Bone marrow-derived macrophages (BMDMs) were stimulated in non-adherent plates for 24 h with LTA, *Spneu* 6303, *Spneu* D39Δcps or medium control. (A) Cell viability was assessed by staining with fixable viability dye and measurement by flow cytometry. (B) TNFα protein levels secreted by non-adherent BMDMs. Comparisons between BMDMs from *Stk11*-ΔM and littermate control mice were analyzed using the multiple t-test. ***P < 0.001, ****P < 0.0001.

**Additional file 2: Figure S2.** Bacterial loads in distant organs during pneumonia caused by encapsulated pneumococci. Bacterial loads [colony-forming units (CFUs) per millilitre] in spleen and liver of *Stk11*-ΔM mice and littermate controls 12 and 40 h after intranasal inoculation with approximately 5 × 10^4^ CFUs of *Spneu* 6303*.* Bacterial loads in *Stk11*-ΔM mice were compared with those to littermate controls using the Mann–Whitney *U* test. All comparisons were not significant.

**Additional file 3: Figure S3.** Lung cytokine and chemokine levels during pneumonia caused by non-encapsulated pneumococci. Mice were inoculated intranasally with approximately 1 × 10^8^ CFUs of *Spneu* D39Δcps and levels of inflammatory mediators (TNFα, IL-6, CXCL1, CXCL2 and MPO) were measured in whole lung homogenates 5 h thereafter. Data are shown as bar with mean ± SD representing 7 mice per group. Protein levels of *Stk11*-ΔM mice were compared to littermate controls using the Mann–Whitney U test. *P < 0.05.

## Data Availability

The datasets used and/or analyzed during the current study are available from the corresponding author on reasonable request.

## Abbreviations

AMPK: AMP-activated protein kinase
AMs: Alveolar macrophages
BAL: Bronchoalveolar lavage
BALF: Bronchoalveolar lavage fluid
BMDMs: Bone marrow-derived macrophages
CFU: Colony forming units
CXCL: C-X-C Motif Chemokine Ligand
EDTA: Ethylenediaminetetraacetic acid
IL: Interleukin
Lkb1: Liver kinase-B1
LPS: Lipopolysaccharide
LTA: Lipoteichoic acid
MOI: Multiplicity of infection
MPO: Myeloperoxidase
PBS: Phosphate buffered saline
RPMI: Roswell Park Memorial Institute
*Spneu*: *Streptococcus pneumoniae*
STK11: Serine/threonine kinase 11
*Stk11*-ΔM: Myeloid-specific Lkb1-deficient
TLR: Toll-like receptor
TNFα: Tumor necrosis factor α

## References

[1] World Health Organization (2018). Global health estimates 2016 (deaths by cause, age, sex, by country and by region, 2000–2016; and life expectancy, 2000–2016).

[2] van der Poll T, Opal SM (2009). Pathogenesis, treatment, and prevention of pneumococcal pneumonia. Lancet.

[3] Hussell T, Bell TJ (2014). Alveolar macrophages: plasticity in a tissue-specific context. Nat Rev Immunol.

[4] Deng J, Wen C, Ding X, Zhang X, Hou G, Liu A (2020). LKB1-MARK2 signalling mediates lipopolysaccharide-induced production of cytokines in mouse macrophages. J Cell Mol Med.

[5] Liu Z, Zhang W, Zhang M, Zhu H, Moriasi C, Zou MH (2015). Liver kinase B1 suppresses lipopolysaccharide-induced nuclear factor kappaB (NF-kappaB) activation in macrophages. J Biol Chem.

[6] Otto NA, de Vos AF, van Heijst JWJ, Roelofs J, van der Poll T (2020). Myeloid liver kinase B1 depletion is associated with a reduction in alveolar macrophage numbers and an impaired host defense during gram-negative pneumonia. J Infect Dis.

[7] Wang Q, Chen S, Li T, Yang Q, Liu J, Tao Y (2021). Critical role of Lkb1 in the maintenance of alveolar macrophage self-renewal and immune homeostasis. Front Immunol.

[8] Gan B, Hu J, Jiang S, Liu Y, Sahin E, Zhuang L (2010). Lkb1 regulates quiescence and metabolic homeostasis of haematopoietic stem cells. Nature.

[9] Gurumurthy S, Xie SZ, Alagesan B, Kim J, Yusuf RZ, Saez B (2010). The Lkb1 metabolic sensor maintains haematopoietic stem cell survival. Nature.

[10] Nakada D, Saunders TL, Morrison SJ (2010). Lkb1 regulates cell cycle and energy metabolism in haematopoietic stem cells. Nature.

[11] Zhang Y, Meng Q, Sun Q, Xu ZX, Zhou H, Wang Y (2021). LKB1 deficiency-induced metabolic reprogramming in tumorigenesis and non-neoplastic diseases. Mol Metab.

[12] Hemminki A, Markie D, Tomlinson I, Avizienyte E, Roth S, Loukola A (1998). A serine/threonine kinase gene defective in Peutz-Jeghers syndrome. Nature.

[13] Li TT, Zhu HB (2020). LKB1 and cancer: the dual role of metabolic regulation. Biomed Pharmacother.

[14] Draing C, Sigel S, Deininger S, Traub S, Munke R, Mayer C (2008). Cytokine induction by Gram-positive bacteria. Immunobiology.

[15] Clausen BE, Burkhardt C, Reith W, Renkawitz R, Forster I (1999). Conditional gene targeting in macrophages and granulocytes using LysMcre mice. Transgenic Res.

[16] Weischenfeldt J, Porse B (2008). Bone marrow-derived macrophages (BMM): isolation and applications. CSH Protoc..

[17] de Vos AF, Dessing MC, Lammers AJ, de Porto AP, Florquin S, de Boer OJ (2015). The polysaccharide capsule of *Streptococcus pneumonia* partially impedes MyD88-mediated immunity during pneumonia in mice. PLoS ONE.

[18] de Porto AP, Liu Z, de Beer R, Florquin S, de Boer OJ, Hendriks RW (2019). Btk inhibitor ibrutinib reduces inflammatory myeloid cell responses in the lung during murine pneumococcal pneumonia. Mol Med.

[19] Hoogendijk AJ, Roelofs JJ, Duitman J, van Lieshout MH, Blok DC, van der Poll T (2012). R-roscovitine reduces lung inflammation induced by lipoteichoic acid and *Streptococcus pneumoniae*. Mol Med.

[20] Meijer MT, de Vos AF, Scicluna BP, Roelofs JJ, Abou Faycal C, Orend G (2021). Tenascin-C deficiency is associated with reduced bacterial outgrowth during *Klebsiella pneumoniae*-evoked pneumosepsis in mice. Front Immunol.

[21] Qin W, Liu Z, van der Poll T, de Vos AF (2022). Induction of acute or disseminating bacterial pneumonia in mice and sampling of infected organs for studying the host response to bacterial pneumonia. Bio Protoc.

[22] de Porto AP, Liu Z, de Beer R, Florquin S, Roelofs J, de Boer OJ (2021). Bruton’s tyrosine kinase-mediated signaling in myeloid cells is required for protective innate immunity during pneumococcal pneumonia. Front Immunol.

[23] Knapp S, Wieland CW, van't Veer C, Takeuchi O, Akira S, Florquin S (2004). Toll-like receptor 2 plays a role in the early inflammatory response to murine pneumococcal pneumonia but does not contribute to antibacterial defense. J Immunol.

[24] van der Poll T, Keogh CV, Buurman WA, Lowry SF (1997). Passive immunization against tumor necrosis factor-alpha impairs host defense during pneumococcal pneumonia in mice. Am J Respir Crit Care Med.

[25] Luck JN, Tettelin H, Orihuela CJ (2020). Sugar-coated killer: serotype 3 pneumococcal disease. Front Cell Infect Microbiol.

[26] Liu Z, Dai X, Zhu H, Zhang M, Zou MH (2015). Lipopolysaccharides promote S-nitrosylation and proteasomal degradation of liver kinase B1 (LKB1) in macrophages in vivo. J Biol Chem.

[27] Zemans RL, Matthay MA (2017). What drives neutrophils to the alveoli in ARDS?. Thorax.

[28] Dessing MC, Schouten M, Draing C, Levi M, von Aulock S, van der Poll T (2008). Role played by Toll-like receptors 2 and 4 in lipoteichoic acid-induced lung inflammation and coagulation. J Infect Dis.

[29] Fielding CA, McLoughlin RM, McLeod L, Colmont CS, Najdovska M, Grail D (2008). IL-6 regulates neutrophil trafficking during acute inflammation via STAT3. J Immunol.

[30] Malley R, Henneke P, Morse SC, Cieslewicz MJ, Lipsitch M, Thompson CM (2003). Recognition of pneumolysin by Toll-like receptor 4 confers resistance to pneumococcal infection. Proc Natl Acad Sci USA.

[31] Srivastava A, Henneke P, Visintin A, Morse SC, Martin V, Watkins C (2005). The apoptotic response to pneumolysin is Toll-like receptor 4 dependent and protects against pneumococcal disease. Infect Immun.

[32] Albiger B, Dahlberg S, Sandgren A, Wartha F, Beiter K, Katsuragi H (2007). Toll-like receptor 9 acts at an early stage in host defence against pneumococcal infection. Cell Microbiol.

[33] Wu Q, Jiang D, Minor MN, Martin RJ, Chu HW (2011). In vivo function of airway epithelial TLR2 in host defense against bacterial infection. Am J Physiol Lung Cell Mol Physiol.

[34] Hippenstiel S, Opitz B, Schmeck B, Suttorp N (2006). Lung epithelium as a sentinel and effector system in pneumonia–molecular mechanisms of pathogen recognition and signal transduction. Respir Res.

[35] Percy MG, Grundling A (2014). Lipoteichoic acid synthesis and function in gram-positive bacteria. Annu Rev Microbiol.

[36] Draing C, Pfitzenmaier M, Zummo S, Mancuso G, Geyer A, Hartung T (2006). Comparison of lipoteichoic acid from different serotypes of *Streptococcus pneumoniae*. J Biol Chem.

[37] Hashimoto M, Tawaratsumida K, Kariya H, Kiyohara A, Suda Y, Krikae F (2006). Not lipoteichoic acid but lipoproteins appear to be the dominant immunobiologically active compounds in *Staphylococcus aureus*. J Immunol.

[38] Kang JY, Nan X, Jin MS, Youn SJ, Ryu YH, Mah S (2009). Recognition of lipopeptide patterns by Toll-like receptor 2-Toll-like receptor 6 heterodimer. Immunity.

[39] Zahringer U, Lindner B, Inamura S, Heine H, Alexander C (2008). TLR2—promiscuous or specific? A critical re-evaluation of a receptor expressing apparent broad specificity. Immunobiology.

[40] Schroder NW, Morath S, Alexander C, Hamann L, Hartung T, Zahringer U (2003). Lipoteichoic acid (LTA) of *Streptococcus pneumoniae* and *Staphylococcus aureus* activates immune cells via Toll-like receptor (TLR)-2, lipopolysaccharide-binding protein (LBP), and CD14, whereas TLR-4 and MD-2 are not involved. J Biol Chem.

[41] Tomlinson G, Chimalapati S, Pollard T, Lapp T, Cohen J, Camberlein E (2014). TLR-mediated inflammatory responses to *Streptococcus pneumoniae* are highly dependent on surface expression of bacterial lipoproteins. J Immunol.

