# Supplementary figures and images for: Myeloid liver kinase B1 contributes to lung inflammation induced by lipoteichoic acid but not by viable Streptococcus pneumoniae
Source: Respir Res. 2022 Sep 12;23:241. doi: 10.1186/s12931-022-02168-6 (PMC9465928; doi:10.1186/s12931-022-02168-6)

**A**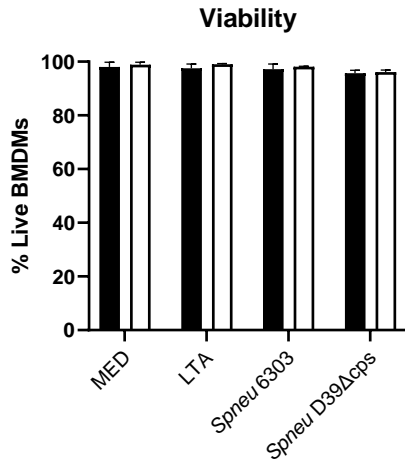**B**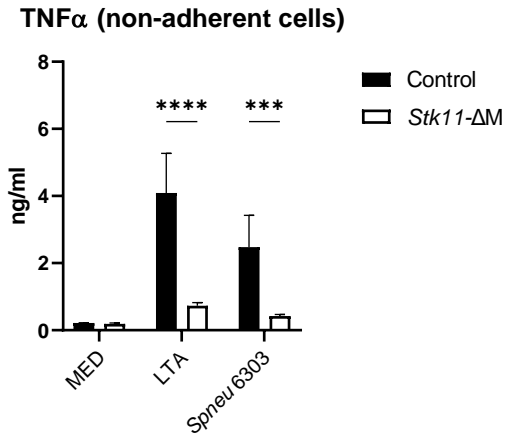

Supplement: Supplementary file 1 — Additional file 1: Figure S1. Viability of in vitro stimulated Lkb1-deficient macrophages. Bone marrow-derived macrophages (BMDMs) were stimulated in non-adherent plates for 24 h with LTA, Spneu 6303, Spneu D39Δcps or medium control. (A) Cell viability was assessed by staining with fixable viability dye and measurement by flow cytometry. (B) TNFα protein levels secreted by non-adherent BMDMs. Comparisons between BMDMs from Stk11-ΔM and littermate control mice were analyzed using the multiple t-test. ***P < 0.001, ****P < 0.0001. [file 12931_2022_2168_MOESM1_ESM.pdf]

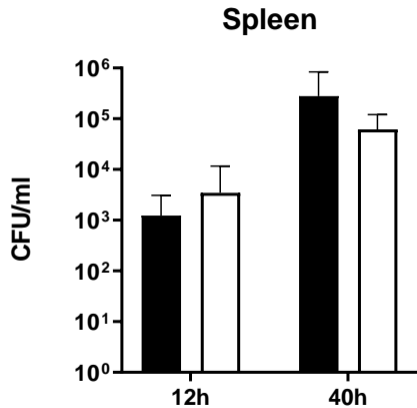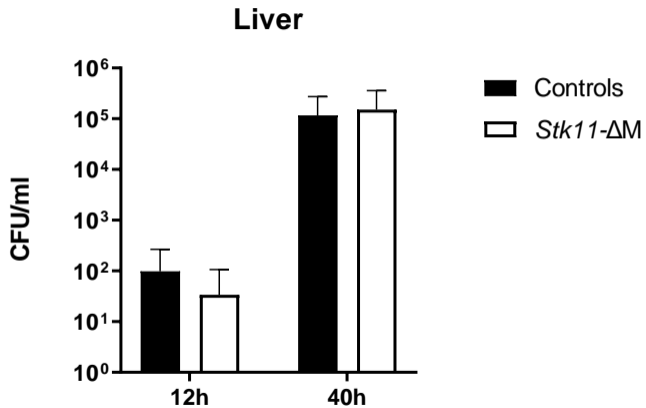

Supplement: Supplementary file 2 — Additional file 2: Figure S2. Bacterial loads in distant organs during pneumonia caused by encapsulated pneumococci. Bacterial loads [colony-forming units (CFUs) per millilitre] in spleen and liver of Stk11-ΔM mice and littermate controls 12 and 40 h after intranasal inoculation with approximately 5 × 10^4^ CFUs of Spneu 6303. Bacterial loads in Stk11-ΔM mice were compared with those to littermate controls using the Mann–Whitney U test. All comparisons were not significant. [file 12931_2022_2168_MOESM2_ESM.pdf]

**TNF $\alpha$** 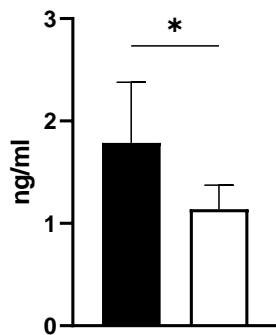**IL-6**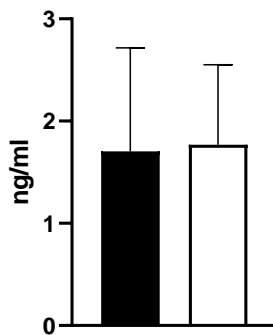

■ Controls  
□ *Stk11*- $\Delta$ M

**CXCL1**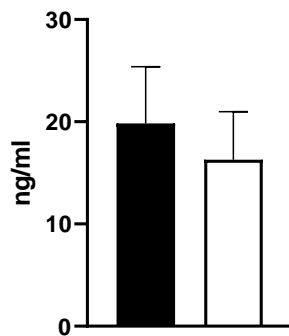**CXCL2**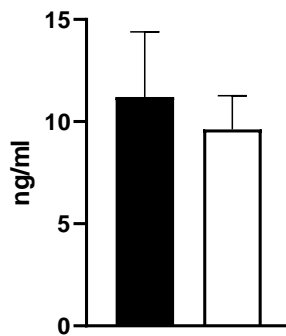**MPO**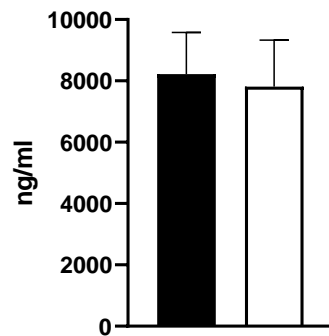

Supplement: Supplementary file 3 — Additional file 3: Figure S3. Lung cytokine and chemokine levels during pneumonia caused by non-encapsulated pneumococci. Mice were inoculated intranasally with approximately 1 × 10^8^ CFUs of Spneu D39Δcps and levels of inflammatory mediators (TNFα, IL-6, CXCL1, CXCL2 and MPO) were measured in whole lung homogenates 5 h thereafter. Data are shown as bar with mean ± SD representing 7 mice per group. Protein levels of Stk11-ΔM mice were compared to littermate controls using the Mann–Whitney U test. *P < 0.05. [file 12931_2022_2168_MOESM3_ESM.pdf]
